# Translation, Adaptation, and Validation of the Swedish Serious Illness Conversation Guide

**DOI:** 10.1177/08258597231210136

**Published:** 2023-10-27

**Authors:** Sofia Andersson, Lisa Granat, Rebecca Baxter, Helene Reimertz, Carina Modéus, Susanna Pusa, Anna Sandgren

**Affiliations:** 1 Center for Collaborative Palliative Care, Linnaeus 8180 University, Växjö, Sweden; 2 Department of Health and Caring Sciences, Linnaeus 8180 University, Växjö, Sweden; 3 Department of Nursing, Umeå University, Sweden; 4 Unit of Palliative Care, Region Kronoberg, Växjö, Sweden

**Keywords:** health communication, palliative care, patients, serious illness conversation, translation, validation

## Abstract

**Objective:** To translate and adapt the Serious Illness Conversation Guide for use within the Swedish healthcare setting and examine the validity and acceptability of the Swedish Serious Illness Conversation Guide. **Methods:** Three rounds of cognitive interviews were conducted (T1-3); patients (T1 n = 11; T2 n = 10; T3 n = 8), family members (T1 n = 5; T2 n = 2; T3 n = 2), and healthcare professionals (T1 n = 6; T2 n = 6; T3 n = 5). The guide was iteratively adapted based on interview feedback, clinical experience, and the literature. The guide was tested on training days with physicians and nurses. **Results:** The Swedish Serious Illness Conversation Guide was found to be useful in supporting serious illness conversations. Clinicians reported that some questions were emotionally challenging. Explicit questions about prognosis and timing were excluded. Instead, the dual approach of “hoping for the best and preparing for the worst” was used to explore patients’ thoughts about the future. **Conclusions:** Patients, family members, and healthcare professionals found the Swedish Serious Illness Conversation Guide to be appropriate, sensitive, and responsive to their needs. The Swedish Serious Illness Conversation Guide may facilitate a more health-promoting approach to serious illness conversations. Further research is needed to understand the impact of these conversations on person-centered and goal-concordant care.

## Introduction

High-quality communication is a cornerstone of care for seriously ill patients who require information in a timely, accurate, and sensitive manner.^1–3^ However, communication about patient goals, values, and preferences frequently comes late in the illness trajectory, which can leave seriously ill patients and their support persons with limited opportunities to influence their care.^1^ The Serious Illness Care Programme (SICP), developed by Ariadne Labs, is a multicomponent intervention that aims to improve the care of persons with serious illness.^1,4^ The SICP is built upon principles of goal-concordant and person-centered care to inform values-based shared decision-making and is comprised of communication tools, clinician training, and systems innovations.^5^ Recent studies found that implementation of the SICP improved the frequency, quality, and timing of serious illness conversations.^5–7^ The program has also been found to have a positive impact on patient anxiety and depression.^8^

The aim of the Serious Illness Conversation Guide (SICG) is to elicit illness understanding, decision-making preferences, share prognostic information according to preferences, understand goals and fears, explore views on trade-offs and impaired function, and wish for family involvement.^4^ While the SICP and SICG were originally developed in English and tested in the American healthcare setting among oncology patients, it has since been adapted for diverse cultural^9,10^ and clinical settings.^11–14^ The SICG has been translated into thirteen languages^15^; however, the processes by which these translations and cultural adaptations occurred, and the acceptability, applicability, and validity of the translated versions have yet to be explored.

During 2017 to 2018, the SICP was adapted and implemented at 2 hospitals in Sweden in partnership with the Center for Collaborative Palliative Care and the Institute of Palliative Care. The project was called “SICP—The Kronoberg Model (1.0).” During the implementation, the SICG was translated (but not validated) and used by specialist physicians. After the implementation, the need to expand the SICP to include nurses was highlighted, with a focus on team collaboration and sustainable organizational structures.^16,17^ To make the SICP suitable for the Swedish context, it is necessary to translate, adapt, and validate the SICG in Swedish. This study therefore aimed to translate and adapt the SICG for use within the Swedish healthcare setting and examine the validity and acceptability of the Swedish SICG.

## Methods

### Study Design

**Figure 1.**
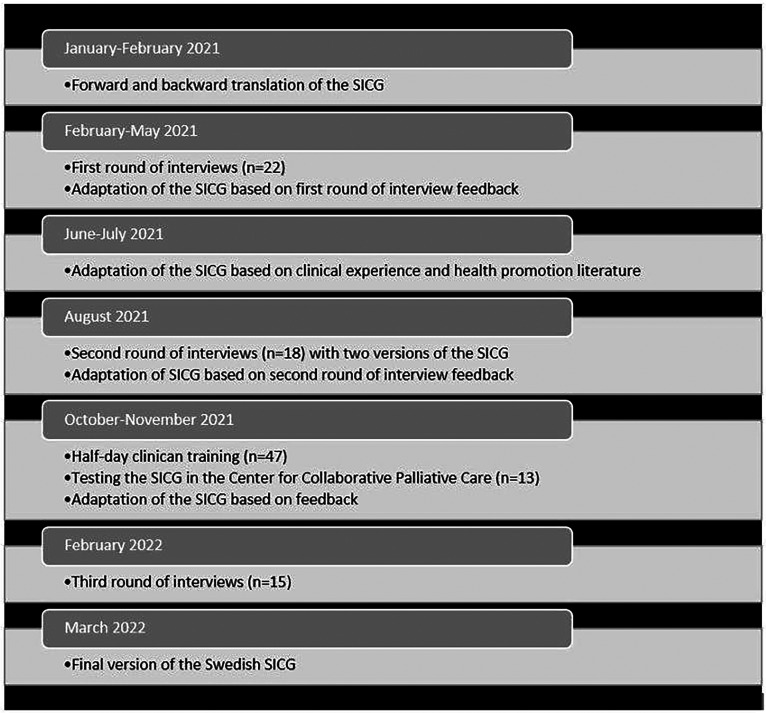
The translation, adaptation, and validation process for the Swedish Serious Illness Conversation Guide (SICG).

The translation, adaptation, and validation of the SICG (version SICG 2017-04-18) was part of an overall project called the “Serious Illness Care Program—The Kronoberg Model (2.0).” Permission was obtained from the original developers at Ariadne Labs, USA.^15^ The translation and adaptation followed Ariadne Labs’ guidelines. An overview of the study process is shown in Figure 1.

### Setting and Participants

The interviews were conducted with patient representatives, family representatives and healthcare professionals (Table 1). A purposive sample of patient and family representatives were recruited from a network in Region Kronoberg and a network Region Cancer Centre South. The contact person for each of the networks for patients with cancer and family members first approached patient and family representatives. If interested, the researchers contacted participants via email and gave information about the study. The inclusion criteria were patients who had, or have had, a life-threatening illness (ie, cancer or neurological diseases), and family members who were related to a patient with a life-threatening illness. Participants were over 18 years old. Healthcare professionals were recruited through the hospital network and, if interested in participating, were contacted by the researchers via email. Healthcare professionals were eligible for inclusion if they were aged over 18 years and had experience in caring for patients with life-threatening illnesses.

**Table 1.** Participants, Age, Gender, Interview Round, and Duration of Interviews.

Participants (n)	Age Mean (min-max)	Gender Female/male	Round 1	Round 2	Round 3
Patient (11)	61 (40-73)	7/5	11	10	8
Family member (5)	65 (46-86)	3/2	5	2	2
Registered nurse (5)	47 (33-58)	3/2	5	4	4
Enrolled nurse (1)	56	1/0	1	1	1
Physician (1)	44	0/1		1	
Duration in minutes—mean (range)			50 (23-75)	32 (16-55)	20 (6-42)

**Table 2.** Domains in the Original SICG (n = 7) and Domains in the Swedish SICG (n = 6).

Original SICG domains	Swedish SICG domains
1) Set-up the conversation	1) Set-up the conversation
2) Assess understanding and preferences	2) Explore the patient's current situation
3) Share prognosis	3) Explore the patient's thoughts about the future from 2 perspectives^a^
4) Explore key topics	
5) Close the conversation	4) Conclude the conversation
6) Document the conversation	5) Document the conversation
7) Communicate with key clinicians	6) Communicate with the care team

^a^ One question with scripted language for physicians and nurses.

### Data Collection and Analysis

#### Step 1: Forward and backward translation

The SICG was forward translated by the first author (SA) and a translation agency into Swedish. The first and last authors (SA, AS) compared the translated and original versions, as well as the translated, nonvalidated version of the SICG which was used during The Kronoberg Model 1.0. The first and last authors both speak Swedish as a first language, have high school English qualifications, have experience in publishing research in English, and are registered nurses with extensive clinical and research experience in palliative care. The SICG was back translated into English by a professional translator. The first and last authors compared the original English SICG with the Swedish forward translation, alongside the English backward translation of the Swedish version, to identify and correct unclear words/sentences.

#### Step 2: First round of interviews and adaptation

Cognitive interviews^18^ were undertaken by 2 authors (SA, LG) and audiotaped. SA conducted 31 interviews, and LG conducted 24 interviews. The interviews were conducted face-to-face (home, hospital) and digitally due to the COVID pandemic and social distancing regulations. Most interviews took place individually (ie, one-on-one), except for 2 interviews which were undertaken with a patient and family member as per their preference. The interview started with questions about participants’ age, gender, employment, education, and experience with palliative care. To facilitate discussion of the SICG, the guide was split into 7 domains: 1) set-up the conversation, 2) assess understanding and preferences, 3) share prognosis, 4) explore key topics, 5) close the conversation, 6) document the conversation, 7) communicate with key clinicians. Participants described their sense of each domain as a whole and were asked whether the text was clear, understandable, relevant, and sensitive. Further questions were asked about whether participants found the text emotional or provocative. They were also asked whether there were specific words that stood out to them, or if they had suggestions for rewording the text or improving the translation. The participants were also asked if they thought the SICG was appropriate for the Swedish healthcare setting. All interviews were summarized by 2 authors (SA, LG) according to each domain and discussed within the author group. In addition to the authors (SA, AS) described previously, the author group also included a political scientist and doctoral student (LG), 2 registered nurses (RB, SP) with clinical experience and doctoral qualifications, and 2 specialist palliative care physicians (HR, CM) with experience in serious illness conversation training.

#### Step 3: Adaptation from clinical experience and literature

Two authors (HR, CM) further adapted the SICG based on their knowledge, specialist clinical experience, and health promotion literature.^19–21^ The importance of honesty, empathy, hope, and preparation in health-promoting communication was discussed and reflected upon in relation to the Swedish healthcare context. These ideas were integrated into the guide adaptation.

#### Step 4: Second round of interviews and adaptation

The second round of interviews were conducted (Table 1) where 2 versions of the Swedish SICG were compared and discussed. Based on the feedback from these interviews, further adjustments were made to the SICG by HR, CM, and AS.

#### Step 5: Half-day training, testing, and adaptation

Physicians (n = 13) and nurses (n = 24) were offered a half-day training in using the Swedish SICG. The training involved an introduction to serious illness conversations, information about how to use the SICG, and practice in using the SICG to have a serious illness conversation. During the training, the physicians and nurses gave verbal feedback regarding how they experienced using the guide and using it in practice. After the half-day training, the author group had several meetings to further discuss the core elements and guiding principles for serious illness communication. The adapted SICG was subsequently presented within the Center X (identifying information). The members of Center of Collaborative Palliative Care practiced using the guide in pairs to test the proposed Swedish SICG for language, readability, and flow (registered nurses, n = 3; enrolled nurse, n = 1; physicians, n = 2; researchers, n = 7).

#### Step 6-7: Third round of interviews and final version of Swedish SICG

After the final changes to the SICG, the third round of interviews were conducted (Table 1). This time, the SICG was sent to the participants before the interviews. The results were discussed among the authors and no further changes were made.

### Ethical Considerations

The study was conducted according to the guidelines of the Declaration of Helsinki.^22^ According to The Act concerning the Ethical Review of Research Involving Humans^23^ and the Swedish Ethical Review Authority guidelines,^24^ this study did not collect sensitive personal data that could be traced back to an individual, and the instrument was not tested on patients, families, or uniformed healthcare professionals. Swedish law and praxis considers this study to be part of normal clinical improvement procedures,^24^ thus approval by an ethics committee was not necessary. All participants were fully informed of the study aim and provided verbal consent. Reasons for nonparticipation were not explored.

## Results

### Step 1: Forward and Backward Translation

The Swedish translation was kept as close to the English SICG as possible. The Swedish version was then back-translated into English. The original English SICG was compared with the Swedish and the Swedish-English versions and adjusted to better fit the Swedish context and vernacular.

### Step 2: First Round of Interviews and Adaptation

The Swedish SICG (version 1) questions were identified by participants as relevant and important, but could also be difficult to discuss since some questions evoked emotions. Some participants reacted to certain words and suggested substitutions. For example, the word “believe/faith” (Swedish: tro) was viewed as being connected to religion. This word was instead changed to the more neutral word “perceive” (Swedish: uppfatta). The word “ability” (Swedish: förmåga) was said to be difficult to understand as it was viewed as being a skill or something that a person was good at. The phrasing was therefore changed to reflect the overall intention of enquiring about things that are especially important for the patient to be able to do.

### Step 3: Adaptation From Clinical Experience and Literature

A second version of the Swedish SICG (version 2) was developed with less focus on sharing prognosis from the perspectives of time, function, and uncertainty. The focus was shifted to a dual approach which emphasized the importance of first listening to the patient's hopes and fears about the future and then asking if the patient wanted to know the healthcare professionals’ opinion. Domains 2 and 3 were combined into: Explore the patient's current situation and their thoughts about the future from 2 perspectives, to hope for the best and prepare for the worst.

### Step 4: Second Round of Interviews and Adaptation

In the second interview round, the 2 versions of the Swedish SICG were compared (Supplementary File 1). Version 1 was described to have more focus on sickness and healthcare, while version 2 was described as more understandable and empathetic. Participants found version 2 more appealing as it used more everyday language to describe concepts and focused on supporting as well as caring for the patient.

*Suggestions for change, to offer support not just care… sometimes you may need support to cope with treatment [patient 9].*

*Spontaneously, this one (version 2) felt more human. I don't know how to explain it, but the other felt more of a sickness perspective. This one (version 2) felt more like you're talking from a friend's perspective. I don't know how to explain it. More like everyday chat. [patient 5]*

Based on this feedback, further adaptations were made to decrease the focus on sickness, prognostication, and timing and instead focus on “hoping for the best and preparing for the worst.” This dual approach still included prognosis, but the word prognosis was not explicitly used in the guide. These changes reduced the number of domains in the Swedish SICG to 6 (Table 2).

**Table 3.** Comparison of Main Changes to the Original SICG (Domain 2-4) and the English Version of the Swedish SICG (Domain 2-3).

Domain/conversation flow	Original SICG	Domain/conversation flow	English version of the Swedish SICG
**2. Assess understanding and preferences**	*“What is your understanding now of where you are with your illness?”* “*How much information about what is likely to be ahead with your illness would you like from me?”*	**2. Explore the patient's current situation** The patient's understanding of their illness What makes the patient feel good What is important for the patient	“*Do you want to share what you think about your illness now?”* “*What makes you feel good in your everyday life?”* “*What is important for you right now?”* “*During difficult periods in your life what has given you strength/energy/power?”*
**3. Share prognosis** Share prognosis Frame as a “wish…worry,” “hope…worry” statement Allow silence, explore emotion	“*I want to share with you my understanding of where things are with your illness…”* Uncertain: “*It can be difficult to predict what will happen with your illness. I hope you will continue to live well for a long time but I’m worried that you could get sick quickly, and I think it is important to prepare for that possibility.”* OR Time: “*I wish we were not in this situation, but I am worried that time may be as short as ___”* (express as a range, eg days to weeks, weeks to months, months to a year). OR Function: “*I hope that this is not the case, but I’m worried that this may be as strong as you will feel, and things are likely to get more difficult.”*	**3. Explore the patient's thoughts about the future from 2 perspectives** Hope for the best Prepare for the worst Worries and fears Need for information about the illness trajectory The focus of care Family Spokesperson Offer Family Guide	“*Can we change focus and talk a moment about the future from two perspectives: To hope for the best and prepare for the worst.”* “*How would you feel if we talk about that?”* “*What do you hope for?”* “*What would it mean to you if that happened?”* “*If we are also to prepare for the worst*—*what do you think is the worst that can happen?”* “*Is there anything you are worried about?”* Physician: “*Do you want to know what I think about your illness now and in the future?”* “*No one can know for sure, but I think…”* “*Take advantage of the time you have and do not wait if there is something that needs to be said or done.”* Nurses: “*Do you want to know what I think about your health now and in the future?”* “*Do you want more information about your illness?”* “*Is there any care you do not want to receive?”* “*Is there anything that is especially important for you to be able to do?”* “*Have you talked to your family about this?”* “*If you could not speak for yourself or make decisions about your care, who would do it for you?”*
**4. Explore key topics** Goals Fears and worries Sources of strength Critical abilities Tradeoffs Family	“*What are your most important goals if your health situation worsens?”* “*What are your biggest fears and worries about the future with your health?”* “*What gives you strength as you think about the future with your illness?”* “*What abilities are so critical to your life that you can’t imagine living without them?”* “*If you become sicker, how much are you willing to go through for the possibility of gaining more time?”* “*How much does your family know about your priorities and wishes?”*		

### Step 5: Half-day Training, Testing, and Adaptation

During the training, some physicians and nurses found it challenging to ask the question “Do you want to know what I think about your illness” since it was connected to prognosis. Therefore, the question was reformulated to address prognosis more generally and include scripted language for physicians and nurses. Phrases for physicians were added, for example “Do you want to know what I think about your illness now and in the future?” Phrases for nurses were also added, such as “Do you want to know what I think about your health now and in the future?” Instructional text for clinicians was inserted at the top of the guide, for example “Only give the patient information they want to have. Listen to the patient's answers. Allow silence. Respond to emotions.” The main differences between the original SICG (domains 2, 3, and 4) and the Swedish SICG (domains 2 and 3) are shown in Table 3. Researchers and clinicians from the Center of Collaborative Palliative Care who practiced using the proposed Swedish SICG did not recommend any further adjustments.

### Step 6: Third Round of Interviews

The final version of the Swedish SICG was tested in a third round of interviews (Table 1), in which participants reported confidence in the Swedish SICG. Participants felt it provided good support for healthcare professionals and described it as being clear, relevant, well-balanced, and complete.

*The seriousness comes through somehow, but it does so in a soft way, I would like to say. It will not be that hard. It is sensitive even though they are clear and relevant issues. // But I think the questions are well formulated so that you, as a patient and a relative, can set the limit yourself as to how much I want to know, how much I want to share… [patient 5].*

### Step 7: Final Version of the Swedish SICG

The SICG template was adapted to the Center of Collaborative Palliative Care by changing the colors and inserting the logo. The Swedish SICG (Supplementary File 2) was subsequently translated into English (Swedish-English SICG, Supplementary File 3) to be used in the Swedish healthcare context for English-speaking persons. The Swedish SICG and the Swedish-English SICG were sent to Adriane Labs for dissemination and are accessible on their webpage.

## Discussion

The most prominent changes to the Swedish SICG included highlighting the dual approach of “hoping for the best and preparing for the worst” and deepening the health-promoting perspective. A salutogenic approach was imbedded during the adaptation, with a focus on well-being, strengths and resources in both the language and questions in the guide. This included changes to the overall domains, conversation flow, and suggested phrases. Added phrases in the Swedish SICG included: *What makes you feel good in your everyday life?* and *What is important for you right now?* This exemplifies the shift away from medicalized assessment to instead exploring the patient's current situation by asking them to describe their lives in their own words. By heightening the person-centered and salutogenic perspective in the Swedish SICG, health-promoting discussions, and reflections regarding strengths, well-being, and values can be facilitated.

The dual approach of “*hoping for the best and preparing for the worst*” is useful in palliative care discussions as it enables simultaneous exploration of both living and dying perspectives.^20^ This dual approach has previously been used in the context of serious illness conversations when discussing illness trajectory and giving information about prognosis.^25–30^ Using positive and negative framing can support the clinician in conveying prognostic information^26,30^ as well as support patients’ prognostic awareness.^29^ Patients are often receptive to the dual approach^31^ preferring phrases with hope/worry statements when discussing prognostic information.^32^ Importantly, consideration of the patient's need for prognostic information is shown by asking if the patient wants to know what the clinician thinks about their illness (physicians) or health (nurses) now and in the future.

This dual approach can support patients and clinicians to reach common understandings^20^ and allows for “big picture” serious illness conversations.^11,33^ It also allows clinicians to explore patients’ illness understanding and discuss health and illness at a pace that aligns with patient preferences.^4^ However, it is important to be aware that each patient has their own coping timeline and might not be ready or willing to discuss certain subjects,^34^ thus the clinician can encourage but not impose the dual approach of hoping and preparing.^20^ In the Swedish SICG patient autonomy is considered by first presenting the dual approach and then asking the patient “*how would you feel if we talk about that?*” This is also relevant for family members, as surrogates have expressed their preference for clinicians to gently support them when facing bad news.^35^ The language regarding the topic of prognosis varies in different adaptations and versions of the SICG.^15^

The Swedish SICG reflects the spirit of the original SICG as expressed by Ariadne Labs^15^ by using open-ended, patient-centered, compassionate language. Language was selected that was appropriate and comfortable for clinicians to use to enhance comprehension. Domain 3 included scripted language with suggested phrases specific for physicians and nurses. According to the Swedish Health laws^36^ physicians are responsible for sharing prognosis according to patients’ information preferences. However, nurses play a significant role in patient care and are therefore well suited to talk about illness and health trajectories. In recent years, the SICG has been adapted for use in different healthcare settings and by a variety of healthcare professionals.^37^ This study only included physicians and nurses; however, it is possible for other professionals such as social workers, chaplains, and assistant nurses to engage in serious illness conversations.^37^ This highlights the need for future studies to explore the use of the Swedish SICG among other professions.

### Strengths and Limitations

A strength of this study is that changes were made based on interview feedback from patients, family members and healthcare professionals. Changes were also made based on current clinical experience and health promotion literature. The Swedish SICG was used in training by physicians and nurses which informed the final version. This consultation process is also recommended by Adriane Labs.^38^ All authors were female and acknowledged that they had preunderstandings informed by their clinical and nonclinical experience, as well as their existing knowledge of the SICP/SICG and palliative care practice area. A user-centered design was used that balanced the need for rigor and structure with the needs of patients, family members, and healthcare professionals.

## Conclusions

The Swedish SICG was appropriate, sensitive, and responsive to the needs of patients, family members, and healthcare professionals. Using the dual approach of hoping for the best and preparing for the worst facilitated discussion of important subjects related to serious illness. The Swedish SICG may facilitate a more health-promoting approach to serious illness conversations. Further research is needed to understand the impact of serious illness conversations on person-centered and goal-concordant care, as well as use of the Swedish SICG in other contexts.

## Supplemental Material

sj-docx-1-pal-10.1177_08258597231210136 - Supplemental material for Translation, Adaptation, and Validation of the Swedish Serious Illness Conversation Guide Click here for additional data file. Supplemental material, sj-docx-1-pal-10.1177_08258597231210136 for Translation, Adaptation, and Validation of the Swedish Serious Illness Conversation Guide by Sofia Andersson, Lisa Granat, Rebecca Baxter, Helene Reimertz, Carina Modéus, Susanna Pusa and Anna Sandgren in Journal of Palliative Care

sj-tiff-2-pal-10.1177_08258597231210136 - Supplemental material for Translation, Adaptation, and Validation of the Swedish Serious Illness Conversation Guide Click here for additional data file. Supplemental material, sj-tiff-2-pal-10.1177_08258597231210136 for Translation, Adaptation, and Validation of the Swedish Serious Illness Conversation Guide by Sofia Andersson, Lisa Granat, Rebecca Baxter, Helene Reimertz, Carina Modéus, Susanna Pusa and Anna Sandgren in Journal of Palliative Care

sj-tiff-3-pal-10.1177_08258597231210136 - Supplemental material for Translation, Adaptation, and Validation of the Swedish Serious Illness Conversation Guide Click here for additional data file. Supplemental material, sj-tiff-3-pal-10.1177_08258597231210136 for Translation, Adaptation, and Validation of the Swedish Serious Illness Conversation Guide by Sofia Andersson, Lisa Granat, Rebecca Baxter, Helene Reimertz, Carina Modéus, Susanna Pusa and Anna Sandgren in Journal of Palliative Care
