# Supplementary material for: Translation, Adaptation, and Validation of the Swedish Serious Illness Conversation Guide
Source: J Palliat Care. 2023 Oct 27;39(1):21–8. doi: 10.1177/08258597231210136 (PMC10687820; doi:10.1177/08258597231210136)
Supplement: sj-docx-1-pal-10.1177_08258597231210136 - Supplemental material for Translation, Adaptation, and Validation of the Swedish Serious Illness Conversation Guide [file sj-docx-1-pal-10.1177_08258597231210136.docx]

Supplementary file 1. Two versions of the Swedish SICG shown in the second round of interviews

| **Swedish SICG Version 1** | | **Swedish SICG Version 2** | |
| --- | --- | --- | --- |
| **Domain/**  conversations flow | Suggested questions and phrasing | **Domain/**  conversation flow | Suggested questions and phrasing |
| **Sharing prognosis**  •Describe the best and worst possible scenarios  •Share hopes and worries  •Allow silence, explore emotions | *“Can we talk for a moment about the future from two perspectives”*  *“I wish the situation was different, but it is likely that your health may worsen…”*  *“It can be difficult to predict how your illness will develop. I hope you continue to live well, but the illness can worsen quickly and it is important that we prepare for that.”*  *“It can be difficult to predict, but it may be that you have a short time left, such as _____ (describe days, weeks, months, years).”*  *“What do you hope for?”*  *“What would it mean to you if that happened?”* | **Explore the patient’s thoughts about the future from two perspectives**  •Describe the best and worst possible scenarios  •Share hopes and worries  •Allow silence, explore emotions | *“Can we talk for a moment about the future from two perspectives: To hope for the best and prepare for the worst?”*  *“How would you feel if we talked about that?”*  *“What do you hope for?”*  *“What would it mean to you if that happened?”*  *Mirroring: “I hear that you are hoping for…and you are thinking that…”*  *“If we also prepare for the worst - what do you think is the worst that can happen?”*  *“Is there anything you are worried about?”*  *“Is there anything you want to know about your illness and what may happen in the future?”*  *“Is there any care that you do not want to receive?”*  *“Is there anything that is particularly important for you to be able to do?”*  *“Have you talked to your family about this?”*  *“If you could not speak for yourself, who would do it for you?”* |
| **Explore key topics**  •Goals  •Fears and worries  •Sources of strength  •Critical abilities  •Tradeoffs  •Family | *“If your health worsens, what is most important to you?”*  *“Looking to the future, what are your most worried about? What are your biggest worries?”*  *“What gives you strength/energy/power/endurance when you think about the future?”*  *“Is there anything that is particularly important for you to be able to do?”*  *“If your health worsens, how much are you willing to go through for the possibility of more time?”*  *“If you have difficulty speaking for yourself, who would do it for you?”*  *“Have you talked to your family about the things that we have discussed today?”* |  |  |
