# Supplementary figures and images for: Translation, Adaptation, and Validation of the Swedish Serious Illness Conversation Guide
Source: J Palliat Care. 2023 Oct 27;39(1):21–8. doi: 10.1177/08258597231210136 (PMC10687820; doi:10.1177/08258597231210136)

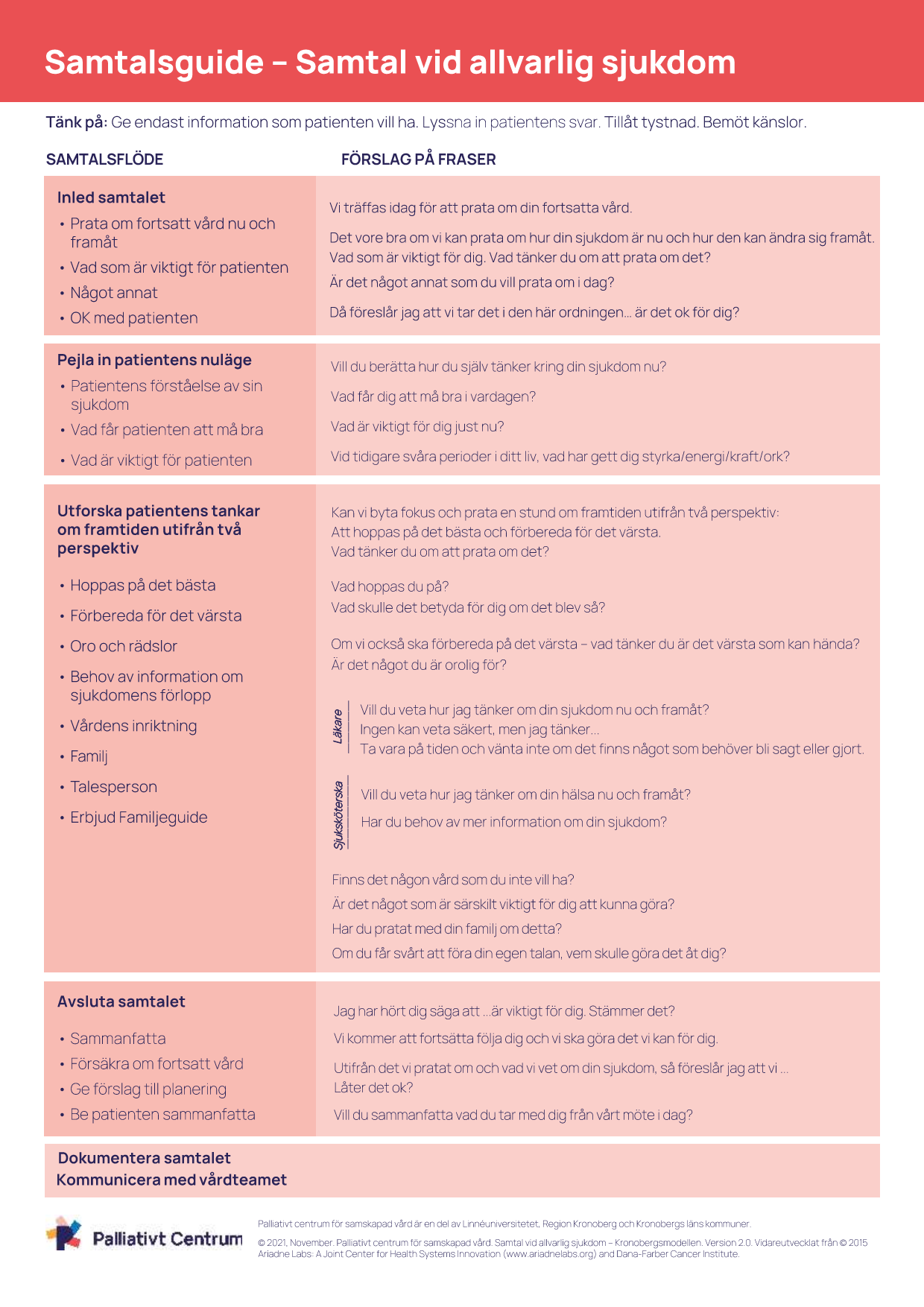

Supplement: sj-tiff-2-pal-10.1177_08258597231210136 - Supplemental material for Translation, Adaptation, and Validation of the Swedish Serious Illness Conversation Guide [file sj-tiff-2-pal-10.1177_08258597231210136.tiff]

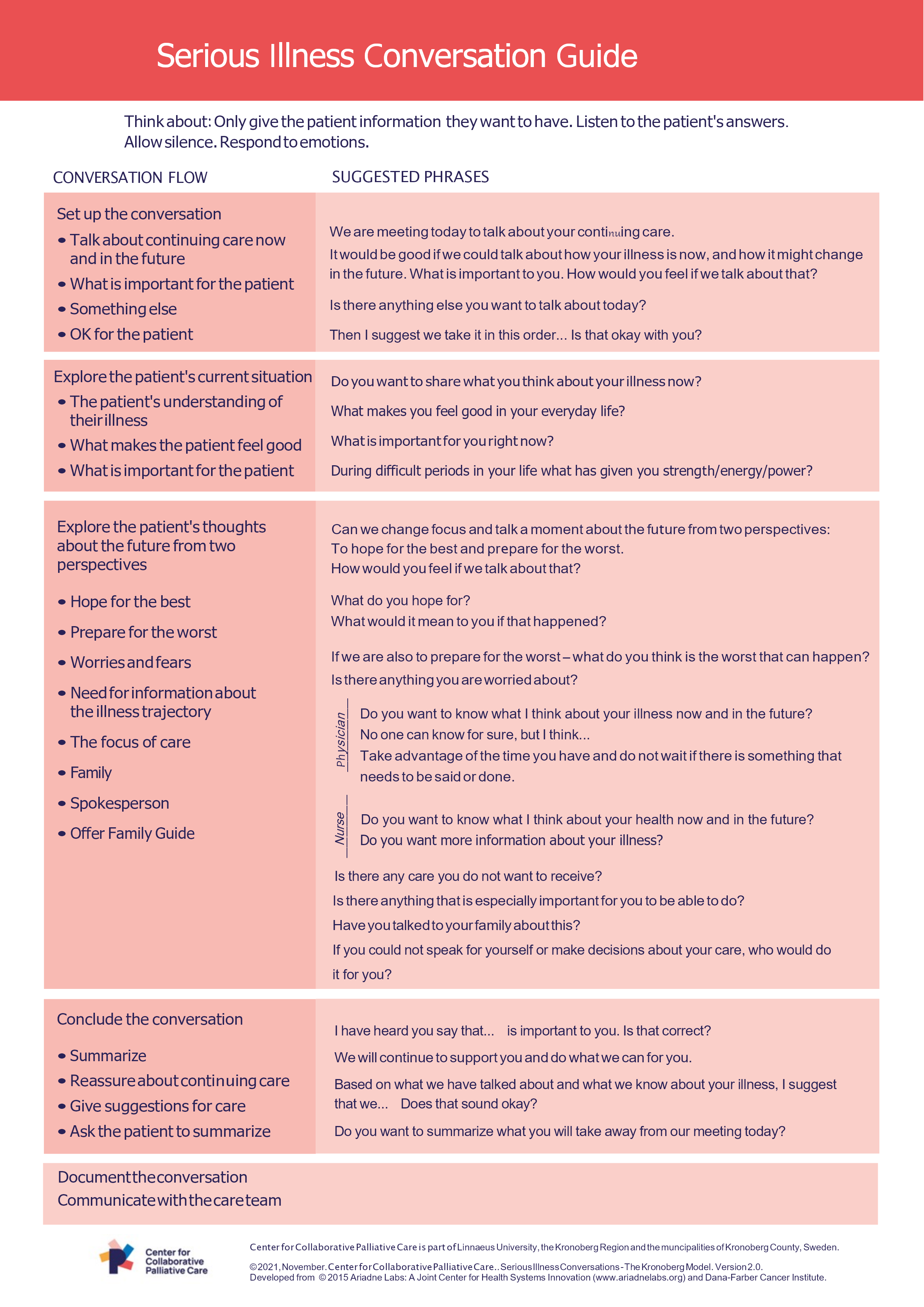

Supplement: sj-tiff-3-pal-10.1177_08258597231210136 - Supplemental material for Translation, Adaptation, and Validation of the Swedish Serious Illness Conversation Guide [file sj-tiff-3-pal-10.1177_08258597231210136.tiff]
